# ‘We get to learn as we move’: effects and feasibility of lesson-integrated physical activity in a Swedish primary school

**DOI:** 10.1186/s12889-024-18509-7

**Published:** 2024-04-19

**Authors:** Robert Larsson, Eva Ljung, Sara Josefsson, Thomas Ljung

**Affiliations:** 1 Division of Public Health Sciences, School of Health, Care and Social Welfare, Mälardalen University, 721 23 Västerås, Box 883, Sweden; 2 Borlänge municipality, Borlänge, Sweden

**Keywords:** Children, Effectiveness, Elementary school, Feasibility, Health, Implementation, Intervention, Movement integration, Physical activity, School

## Abstract

**Background:**

Physical activity (PA) promotes health in adults as well as children. At the same time, a large proportion of children do not meet the recommendations for PA, and more school-based efforts to increase PA are needed. This study investigates the effectiveness and feasibility of lesson-integrated PA in a Swedish primary school.

**Methods:**

We evaluate a new method called ‘Physical Activity and Lesson in Combination’ (abbreviated FALK in Swedish) using a mixed methods approach; a quasi-experimental study followed by qualitative interviews. Two schools participated in the study, one constituting the intervention group (I-school, *n* = 83) and the other the control group (C-school, *n* = 81). In addition to regular physical education, the I-school had three 30-minute FALK lessons each week. A total of 164 students aged 7–9 years wore pedometers for a whole week, four times over two semesters, and the number of steps per day (SPD) and the proportion of students with < 10,000 SPD were compared. Statistical differences between the schools were tested with ANOVA, Chi2, t-tests, and ANCOVA. Interviews with students (*n* = 17), parents (*n* = 9) and teachers (*n* = 9) were conducted and analysed using qualitative content analysis.

**Results:**

The results show that FALK led to the I-school getting more SPD and fewer students with < 10,000 SPD. Also, FALK was experienced as a positive, clear, and flexible method, simultaneously encouraging PA and learning. Challenges experienced concerned the teachers’ work situation, time, finding suitable learning activities, outdoor school environment changes, and extreme weather conditions.

**Conclusions:**

This study indicates that FALK has the desired effects on PA and is a feasible method of integrating PA into theoretical teaching. We conclude that FALK is worth testing at more schools, given that implementation and sustainment of FALK considers both general enablers and barriers, as well as context-specific factors at the individual school.

**Supplementary Information:**

The online version contains supplementary material available at 10.1186/s12889-024-18509-7.

## Background

In recent years, there has been a stream of reports and surveys showing that children and young people have limited physical activity (PA) [1, 2]. For example, a Swedish study shows that only 43 per cent of adolescent boys and 23 per cent of girls of the same age meet the World Health Organisation (WHO) recommendations of engaging in at least 60 minutes of PA a day of moderate to vigorous intensity [3]. Physical inactivity is a well-known risk factor for ill health and disease; at the same time, there is strong scientific evidence for PA and its health-promoting and preventing effects among children and adolescents [4, 5]. Additionally, previous research shows that PA can have positive effects on cognitive abilities and academic achievements [6, 7, 8].

As children spend a lot of time in school, it is an important health-promoting arena and a supportive environment for developing both positive health-related behaviours and learning [9]. The school is also important for health equity given that physical inactivity is more common among families and children where the parents have lower education and socioeconomic status [3, 10].

Previous research shows that school-based health interventions focusing on PA can have beneficial effects on physical and mental health among children and adolescents [3, 11]. Some research has focused on increasing PA during physical education lessons [12]. At the same time, it does not seem to be enough to increase PA during physical education lessons; school children also need to increase PA outside physical education lessons to increase their total level of PA.

Both internationally and in Sweden, various projects and studies have explored new ways to increase PA *before*, *during* and *after* school. A well-known Swedish example is the Bunkeflo project, which aimed to increase the daily PA among school children [13]. Other initiatives deal with lesson-integrated PA (also called movement integration), which incorporates PA, at any intensity level, within normal classroom education and in other school subjects than physical education [14]. Among these initiatives, there is a wide range of activities including active lessons and active breaks [14, 15]. Research shows that active lessons can have positive effects on both PA and academic achievement [8, 16]. However, there are also challenges with implementing active lessons and lesson-integrated PA in primary schools. One challenge is the limited time for physical education in the curriculum, and conducting PA within other lessons can create tensions as lesson-integrated PA can be perceived as stealing valuable time from teaching the subject. Previous research shows several enablers and barriers when lesson-integrated PA is implemented in primary schools. In a systematic review by Michael et al. [17], teachers’ motivation and self-confidence together with organisational support, time and resources are crucial factors. However, there is a need for further research about *what* effects can be expected under real-world circumstances (effectiveness) and *how* lesson-integrated PA works in practice. The latter involves the need for in-depth knowledge about the feasibility and implementation of lesson-integrated PA [18].

The present study investigates the effects and experiences of a new method integrating PA into theoretical teaching. The method is called ‘Physical Activity and Lesson in Combination’ (abbreviated FALK in Swedish; hereafter we use the Swedish abbreviation) [19, 20]. FALK is intended to encourage students to be physically active, and to practice pulse-raising activities during theoretical teaching in all subjects. The overall goal of FALK is to develop a pragmatic method for increased total PA among students. Thereby, the FALK method follows calls for pragmatic approaches in public health research, meaning interventions focusing on issues and information relevant to decision-making and action-taking, and balancing results relevant to stakeholders without abandoning scientific rigour [21]. Consequently, the study aims to investigate the effectiveness and feasibility of lesson-integrated PA in a primary school. The following research questions are explored:

1. To what extent does lesson-integrated PA affect the students’ total PA?
2. How are enablers and barriers experienced by students, parents and teachers when conducting lesson-integrated PA?
3. What improvements regarding lesson-integrated PA do students, parents and teachers identify?

## Methods

A mixed methods approach was used to investigate the effects of FALK and the experiences of lesson-integrated PA. More specifically, the study used an explanatory sequential mixed methods design [22] in which a quasi-experimental study of PA effects was followed by qualitative interviews, focusing on experiences of FALK under real-world conditions in a primary school setting.

### Intervention characteristics and research setting

The goal of FALK was to develop a pragmatic method that increases student’s total level of PA by integrating PA in ordinary lessons, thereby achieving lesson-integrated PA (i.e. FALK lessons; see Additional file 1). The intervention used a quasi-experimental design with students in the intervention school completing three FALK lessons for 30 minutes a week, in addition to regular physical education lessons (two 40 minute lessons per week). Students from another primary school served as a control group and participated in regular physical education (two 40 minute lessons per week). PA was the primary outcome measure of the intervention and was objectively measured using pedometers.

In the present study, the FALK intervention was conducted with students in grades 1 to 3 (7–9 years old) at a municipal primary school located in a small community outside a medium-sized city in Sweden. The intervention took place during the academic year, in the autumn of 2020 and spring of 2021. Before the FALK intervention began the principal gave her approval and support. Two teachers (SJ and EL), who had participated in a pilot study, informed all teachers at both the intervention and control school (I-school and C-school) about FALK, and at the I-school, a total of twelve teachers conducted FALK lessons. Several FALK lessons, and related work materials, had already been prepared from the pilot study. Thereafter, more FALK lessons and work materials were developed, in preparation for the start of FALK at the intervention school. The FALK study was approved by the Swedish Ethical Review Authority in Stockholm (dnr 2020 − 00922).

### Participants

In the quasi-experimental study, students from two primary schools were recruited (7–9 years old) and with one school’s students participating in the FALK intervention (i.e. intervention group, I-school) and the other school’s students acting as the control group (C-school). A total of 164 students participated (see Table 1).

**Table 1:** Distribution among the two schools of participating students regarding grade and girls/boys

	FALK intervention I-school	Control C-school	Sum
Grade 1	Girls: 11 Boys: 9	Girls: 17 Boys: 11	Girls: 28 Boys: 20
Grade 2	Girls: 9 Boys: 14	Girls: 16 Boys: 12	Girls: 25 Boys: 26
Grade 3	Girls: 22 Boys: 18	Girls: 14 Boys: 11	Girls: 36 Boys: 29
**Sum**	Girls: 42 Boys: 41	Girls: 47 Boys: 34	**Girls: 89** **Boys: 75**

The I-school and C-school are located a few kilometres apart in a rural community outside the city. The two schools showed no major differences in terms of lesson content, outdoor school environment or student living conditions. The same principal is responsible for both schools, the teachers at the two schools have common planning of the educational content and the students at both schools engage in the same kind of leisure activities both during warm and cold seasons. Therefore, we consider the risk of selection bias to be small. In more detail, the groups at the C-school and I-school participated in the FALK study as follows:

#### C-school (control group)

Regular physical education lessons twice a week for 40 minutes, plus the possibility of voluntary or organised recreational activities with physical movement.

#### I-school (intervention group)

In addition to regular physical education lessons, and the possibility of voluntary or organised recreational activities with physical movement, three FALK lessons of 30 minutes each were carried out continuously every week over two semesters (i.e. one academic year). Class teachers, leisure leaders and/or physical education teachers organised and carried out the FALK lessons based on the curriculum for each grade and the student’s knowledge levels and maturity.

The qualitative interview study involved three groups of participants: students, parents (guardians), and teachers. Purposeful sampling was used to select the participants based on their experience of FALK [23]. In practice, the sampling was made by selecting students who had participated in FALK lessons, parents of students participating in FALK lessons, and teachers responsible for conducting FALK lessons.

### Data collection

The quantitative data collection was conducted using a pedometer (Yamax LS2000/SW200). Students and parents were instructed on how to use the pedometer. All readings and documentation of pedometer data were carried out by staff at the I-school and C-school. The pedometers were worn by students at both schools during all waking hours for seven consecutive days on four measurement occasions:

1. Sep-20 (week 37) immediately before the start of the intervention (baseline measurement).
2. Nov-20 (week 46) at the end of the autumn term.
3. Feb-21 (week 6) at the beginning of the spring term.
4. May-21 (week 18) at the end of the intervention.

Qualitative data were collected by semi-structured interviews [24], and in total, 17 students (nine girls and eight boys representing grades 1 to 3), nine parents (five women and four men), and nine teachers (eight women and one man) were interviewed. All interviews were individual, face-to-face, and conducted using an interview guide with open-ended questions. The interview questions were straightforward and focused on what had worked well, less well, and what could be improved in FALK. The interviews were conducted by two of the authors (EL and SJ) and were documented by taking notes. For interviews with students, written informed consent was obtained from parents (guardians) and verbal consent was obtained for interviews with parents and teachers. All interviews were conducted after the intervention (i.e. May-June 2021).

### Analysis

In the study design phase, sample size and power were calculated. Based on a previously conducted pilot study (2018, unpublished), the approximate mean number of steps per day (SPD) was expected to be 11,000 and the standard deviation 3,000. Clinically relevant differences/changes were estimated to be 1,500 SPD (equivalent to, approximately, a one-kilometre walk). Sample size calculations showed that with a statistical power of 80% and α = 0.05, 63 students per group were required.

The statistical analysis began with calculating an average value for the number of SPD for each student. Calculations were conducted for weekdays (Monday morning to Friday afternoon), weekend days (Friday afternoon to Monday morning) and all seven days of the week (total PA of the week). An analysis of variance, specifically a mixed between-within-subjects ANOVA, was conducted to examine differences in total PA (measured as the average number of SPD for the entire week) between schools over time. Independent t-tests were then carried out on each of the four measurement occasions to examine the difference in SPD average values between the I-school and C-school on weekdays, weekends, and for all seven days in the current measurement week. A one-way between-groups analysis of covariance (ANCOVA) was conducted to adjust for the (non-significant) baseline difference in SPD. Finally, we examined the percentage of students at each school who did not achieve 10,000 SPD per day on average for the entire week at each measurement time. These results are presented in cross tables, for all students and girls and boys separately. Differences in these proportions were analysed with the Chi2 test on each of the four measurement occasions. All statistical analyses were conducted using SPSS Statistics version 26.

The interview data were analysed using qualitative content analysis [25]. We started the analysis by reading the interview notes to familiarise ourselves with the data, and thereafter we started the open coding by searching for keywords, phrases, and meaningful sentences. In the open coding process, initial codes were identified and sorted into potential subcategories, which were later collapsed into broader generic categories. After this process, subcategories and categories were reviewed and further refined. The interview material was first analysed inductively and separately for the three interview groups (i.e. students, parents, and teachers) and then brought together to form a holistic picture. All authors were involved in the final stages of the analysis, and the results were discussed to ensure credibility.

## Results

First, the quantitative results are presented, followed by the qualitative results. All results are interpreted and discussed in the discussion section.

### Quantitative results

Differences in PA between the I-school and the C-school are reported here first as SPD, then as the proportion of students with fewer than 10,000 SPD. The analysis of variance showed no significant interaction between measurement time and school, Wilk’s Lambda = 0.93, F(3.83) = 2.06, *p* = 0.11, partial eta squared = 0.07. There was a significant main effect of measurement occasion, Wilk’s Lambda = 0.57, F(3.83) = 20.50, *p* < 0.01, partial eta squared = 0.43. There was also a significant main effect of school, F(1.85) = 4.64, *p* < 0.05, partial eta squared = 0.05. This indicates that FALK contributes to increased total PA. The students included in the analysis of variance and their SPD at each measurement point are presented in Table 2.

**Table 2:** Average number of SPD for all seven days of each measurement week for those students at I-school and C-school whose values were included in the variance analysis

Measurement week (M)	Number of students I-school	SPD I-school students	SD I-school	Number of students C-school	SPD C-school students	SD C-school
**M1** (week 37)	45	13 291	3 521	42	12 816	3 341
**M2** (week 46)	45	11 983	2 614	42	11 187	3 196
**M3** (week 6)	45	12 295	2 206	42	10 817	3 002
**M4** (week 18)	45	14 664	3 651	42	12 536	3 813

SPD = Steps per day; SD = Standard Deviation

**Fig. 1:**
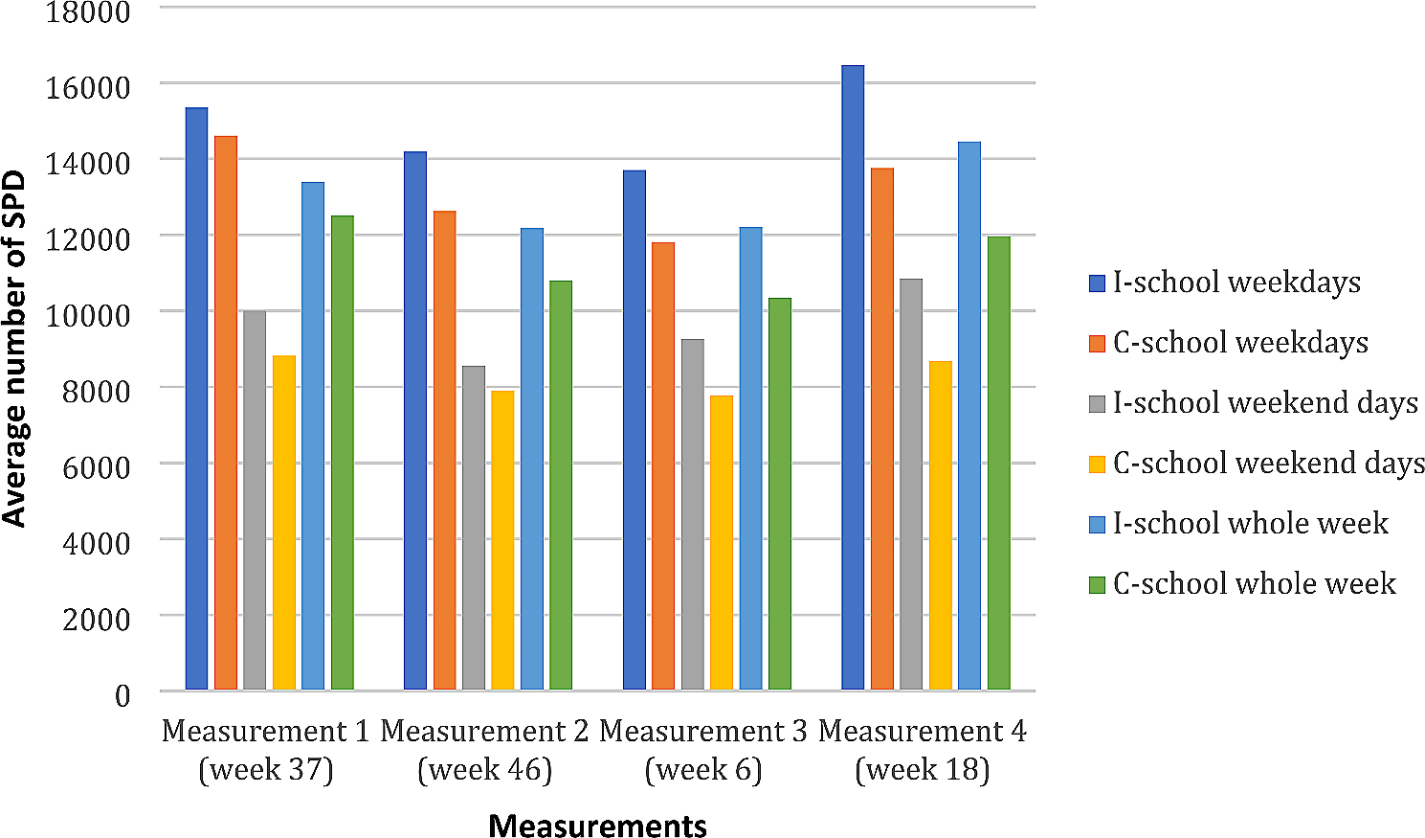
Average number of SPD for students at I-school and C-school at each measurement occasion for weekdays, weekend days and the whole week (SD shown in Table 3)

On weekdays, there was no statistically significant difference in mean SPD at the baseline measurement (i.e. immediately before the intervention), but when the FALK lessons were ongoing (measurements 2 to 4), the I-school had more SPD than the C-school. On weekends, the I-school had more SPD than the C-school at all four measurements, a difference that was statistically significant at measurements 3 and 4. For total PA (‘whole week’), there was no statistically significant difference between the I-school and C-school students at the first measurement, but at the three subsequent measurement weeks, the I-school had more SPD than the C-school (see Table 3; Fig. 1).

**Table 3:** Average number of SPD for weekdays, weekend days and for all seven days of each measurement week for those students at I-school and C-school whose values were included in the analyses with t-test

Measurement week (M)	Number of students I-school	SPD I-school students (SD)	Number of students C-school	SPD C-school students (SD)	Independent t-test (2-tailed)
**M1** Weekdays	73	15 348 (4 331)	66	14 612 (3 471)	n.s. (*p* = 0.27)
**M2** Weekdays	79	14 191 (3 360)	73	12 628 (3 601)	**
**M3** Weekdays	77	13 707 (2 368)	71	11 813 (3 050)	***
**M4** Weekdays	74	16 471 (3 585)	71	13 758 (3 638)	***
**M1** Weekend days	62	10 006 (4 120)	62	8 822 (4 419)	n.s. (*p* = 0.12)
**M2** Weekend days	74	8 552 (4 053)	66	7 894 (5 051)	n.s. (*p* = 0.39)
**M3** Weekend days	74	9 266 (3 036)	75	7 770 (4 185)	*
**M4** Weekend days	72	10 854 (4 062)	69	8 689 (4 773)	**
**M1** Whole week	61	13 393 (3 645)	60	12 507 (3 216)	n.s. (*p* = 0.16)
**M2** Whole week	74	12 174 (2 989)	65	10 800 (3 131)	**
**M3** Whole week	73	12 203 (2 231)	71	10 345 (2 922)	***
**M4** Whole week	72	14 459 (3 289)	69	11 964 (3 469)	***

SPD = Steps per day; SD = Standard Deviation; n.s. = not statistically significant, * = *p* < 0.05; ** = *p* < 0.01; *** = *p* < 0.001

At the first measurement (M1 Whole week, before the start of the intervention), the I-school had an average of 886 more SPDs than the C-school. Although this difference was not statistically significant, one could argue for using statistical methods to adjust for differences between the groups in baseline values. A one-way between-groups analysis of covariance (ANCOVA) was conducted. After adjusting for the difference in pre-intervention SPDs between the groups at M1, there was no longer a statistically significant difference between the groups at M2 (*p* = 0.08) but the statistically significant differences remained at M3 and M4 (*p* < 0,01).

Moreover, girls had fewer SPD on average than boys (Table 4). There was also a higher proportion of girls, compared to boys, who did not reach 10,000 SPD (Table 5). A large proportion of students fell below 10,000 SPD. Before the intervention, the proportion was similar in both schools. During the weeks of measurement when FALK lessons were taking place, the I-school had significantly fewer students with less than 10,000 SPD (Table 5).

**Table 4:** Comparison of all participating girls and boys in terms of average number of SPD for all seven days included in each measurement week

	SPD Girls (SD)	SPD Boys (SD)	Independent t-test
Measurement week 1	12 140 (3 011)	13 963 (3 721)	**
Measurement week 2	10 515 (2 597)	12 952 (3 257)	***
Measurement week 3	10 912 (2 698)	11 729 (2 762)	n.s. (*p* = 0.08)
Measurement week 4	12 461 (3 277)	14 147 (3 750)	**

SPD = Steps per day; SD = Standard Deviation; n.s. = not statistically significant, * = *p* < 0.05; ** = *p* < 0.01; *** = *p* < 0.001

**Table 5:** Number and percentage of students with less than 10 000 steps/day on average for the whole week, described for all students at each school and for girls and boys separately

	I-school	C-school	Chi2-test
Measurement 1 (week 37) All students	11 of 61 (18%)	15 of 60 (25%)	n.s. (*p* = 0.477)
Measurement 2 (week 46) All students	19 of 74 (26%)	30 of 65 (46%)	*
Measurement 3 (week 6) All students	12 of 73 (16%)	39 of 71 (55%)	***
Measurement 4 (week 18) All students	4 of 72 (6%)	25 of 69 (36%)	***
Measurement 1 (week 37) Girls only	7 of 32 (22%)	11 of 35 (31%)	n.s. (*p* = 0.545)
Measurement 2 (week 46) Girls only	16 of 41 (39%)	22 of 40 (55%)	n.s. (*p* = 0.223)
Measurement 3 (week 6) Girls only	7 of 36 (19%)	25 of 42 (60%)	**
Measurement 4 (week 18) Girls only	3 of 36 (8%)	16 of 40 (40%)	**
Measurement 1 (week 37) Boys only	4 of 29 (14%)	4 of 25 (16%)	n.s. (*p* = 1.00)
Measurement 2 (week 46) Boys only	3 of 33 (9%)	8 of 25 (32%)	n.s. (*p* = 0.062)
Measurement 3 (week 6) Boys only	5 of 37 (14%)	14 of 29 (48%)	**
Measurement 4 (week 18) Boys only	1 of 36 (3%)	9 of 29 (31%)	**

n.s. = not statistically significant, * = *p* < 0.05; ** = *p* < 0.01; *** = *p* < 0.001

### Qualitative results

In the analysis, three descriptive categories were created. The categories focus on the students’, parents’, and teachers’ experiences with FALK illustrated with quotes.

#### A new way of working meets students, parents, and teachers

Students, parents, and teachers express positivity about the new way of working that FALK entails. The students experience FALK as rewarding: *“We get to learn a lot of things at the same time as we move”* (student, grade 2). Also, students enjoy the fun aspects and appreciate participating in developing PA exercises. Like the students, parents support FALK for combining movement and learning, expressing a need for increased student movement during school days.

Teachers perceive FALK as clear, flexible, and enhancing the joy of movement and learning. Teachers emphasise that FALK does not need to be complicated, but instead FALK is seen as a flexible method that can be varied based on subject, class size, weather, and season. Also, teachers value that FALK lessons are explicitly integrated into schedules, emphasizing their compulsory nature akin to other subjects.

A positive effect of FALK emphasised by all involved is the calming influence on the classroom after the FALK lesson. Both students and parents say it is positive for students to reduce excess energy, and the teachers emphasise the students’ enhanced educational focus in subsequent lessons.

*“I think they [the students] are calmer after an outdoor lesson. We have good lessons afterwards in the classroom.”* Teacher.

The students talk about challenges in FALK with inattentive classmates creating anxiety when the teacher gives instructions and FALK lessons being less enjoyable when they perceive the learning activities as too difficult. A few teachers also find FALK too ‘controlled’ and struggle with the integration of pulse-raising activities with subject teaching, such as finding the right balance between PA and relevant learning activities.

*“To combine this [traditional lesson] with the fact that it has to be physical activity with increased heart rate… This has become a bit too artificial for me to achieve.”* Teacher.

Teachers can also face challenges in fostering motivation, commitment, and calm during FALK briefings. Despite these challenges, the teachers note that achieving student motivation and calmness during lessons are universal and not exclusive to FALK teaching.

Parents see improved information dissemination as desirable; they wish to know more about FALK and are curious about the results. Parents find FALK inspiring and advocate sharing information with other classes and schools in the municipality.

#### A new way of working meets the school and the teacher’s working day

The new way of working that FALK entails influences *how* the teachers work. Teachers express that FALK foster innovative thinking on combining PA and teaching. FALK encourages collaboration, allowing teachers to share work material, draw inspiration, and create new material together.

*“We have taken turns to make different materials, and it has been rewarding to get new ideas from another colleague.”* Teacher.

Collaboration is also encouraged by two teachers facilitating FALK lessons. For example, tasks can be divided between the teachers, simplifying student reporting of assignments, and making it easier to support students.

A challenge teachers describe is FALK lesson planning, requiring time to adopt the ‘FALK mindset’ and creating work materials for lessons. Also, it can be challenging to introduce substitutes in the FALK way of working if regular teaching staff is absent.

While FALK enhances collaboration, teachers working alone with FALK lessons ask for more cooperation and collegial support. Some teachers suggest better informing on FALK in the teaching team before implementation and call for improved structuring and organisation of work material by grade and subject.

#### The influence of the surrounding school environment

Changes in the surrounding school environment and weather conditions affects FALK implementation. According to students and teachers, a schoolyard rebuilding has occasionally made FALK lessons challenging, with the schoolyard temporarily reduced and having other students in the schoolyard. This posed difficulties for students to concentrate on the FALK lesson due to distractions in the schoolyard.

*“It was tough in the fall when there were several students who couldn’t focus due to various reasons and it made it difficult to be out with many distractions. The rebuilding of the schoolyard has made the work somewhat difficult”.* Teacher.

Concerning the external school environment, teachers suggest a dedicated pre-lesson gathering spot where students can meet before FALK lessons, like the football field or a nearby wooded area. Heavy rain and cold winter days sometimes pose challenges in carrying out FALK lessons as planned. Teachers have on occasions been forced to rethink, leading to indoor PA activities like active breaks as part of regular lessons. Students also express less enjoyment in FALK lessons during wet and cold conditions.

## Discussion

In this study, we investigate the effects of FALK and how students, parents and teachers have experienced its feasibility at a municipal primary school. As far as we know, our study is the first Swedish study exploring lesson-integrated PA in primary schools, and one of just a few European studies investigating movement integration (MI) interventions in a primary school setting using a researcher-teacher collaboration approach [15].

It is recommended that children and adolescents 6–17 years of age should be physically active for at least 60 minutes every day [4]. This equates to just over 10,000 SPD, slightly more for boys than girls [26]. To detect students who are most likely to fall short of the recommendations, this study used an average value of 10,000 SPD for both girls and boys as the upper limit for insufficient PA.

The reason for measuring the number of steps on seven consecutive days (i.e. also on weekends although the FALK lessons were conducted during school hours on weekdays) is that we wanted to measure children’s total PA during the whole week. This is in line with the ActivityStat Hypothesis [27], which states that if you increase your PA in one area (e.g., during school hours), you will compensatively decrease your PA in another area (e.g., during the weekend) to maintain a stable level of total PA (or energy consumption). Therefore, we wanted to know whether increasing PA at school would lead to less PA during the weekend (which would be undesirable).

One might think it is a given that the total PA level will increase if school-based interventions to increase PA are implemented. However, it must be remembered that a small ‘dose’ of increased PA during the school day is still a relatively limited fraction of the total time available to be physically active, or inactive, which again relates to the the ActivityStat Hypothesis [27]. Previous research shows that the majority of MI interventions have a PA dose of 10–20 minutes per day [15], and while interventions can have positive impacts on total PA, there is also variability and uncertainties in results [8, 16]. With this said, the quantitative results show that FALK increased the proportion of students exceeding 10,000 SPD. We see this result as an indication that FALK has a positive and significant effect on PA among students who, for various reasons, are at risk of falling short of the recommended PA level [4]. The ambition of FALK is not for students to become athletes, but rather for the students ‘most in need’ to move enough to reach a minimum level of PA from a health perspective.

It has been shown that school children move less on weekends compared to school days [3, 28], which is consistent with our results. This further underlines the importance of the school as a health-promoting setting enabling PA for all students, making the school setting especially important for those students most in need of increased PA.

Not surprisingly our results show that outdoor temperature appears to impact the SPD among the students. Compared to the first measurement (week 37), the average SPD at both schools decreased at the second and third measurements (week 46 and week 6 respectively) and then increased at the final measurement (week 18). However, there was a large difference between schools in terms of seasonal variation in the proportion of students with less than 10,000 SPD. At the C-school, this proportion increased sharply in late autumn (measurement 2) and winter (measurement 3), while the change in the I-school was comparatively small.

The quantitative results demonstrate significant differences in average SPD between the two schools at the measurements when FALK is ongoing at the I-school, which we believe helps to provide a clearer picture of the effects of FALK. The difference between our two schools in SPD for the ‘whole week’ increases with the duration of the FALK intervention, and we believe that comparison at measurement 4 is the most interesting, as students at the I-school had conducted FALK lessons for almost two full semesters. At the fourth measurement, the difference between the schools was 2,495 SPD and adjusted for baseline values (i.e. the difference of 886 SPD at measurement 1), the difference at measurement 4 is 1,609 SPD. One might think that a difference in total PA of just over 1,600 SPD on average is not very impressive, but the change contributed by FALK seems to have occurred mainly in students with less than 10,000 SPD, which means it can improve health among students most in need.

Several studies have shown that girls are less physically active than boys [2, 3, 10], and we found the same pattern in our study. The FALK intervention did not close the gender gap in total PA, but we did see a step in the closing direction as there was a significant reduction in the proportion of girls at the I-school who did not reach 10,000 SPD. The latter, to some extent, contradicts previous studies suggesting that school-based interventions increase PA and produce the desired results for boys, but not to the same extent for girls [29, 30].

Moreover, the qualitative results are well in line with previous research on enablers and barriers in the implementation of lesson-integrated PA in primary schools [17]. FALK is perceived as a clear and flexible method by teachers, and positive perceptions and ease of implementation of the new method are among the enablers in previous studies [17]. The results that teachers consider FALK as a clear and flexible method is important as one goal of FALK is to provide a pragmatic method for the integration of PA and learning. Another important result is that FALK contributes to the students being calmer in the classroom after FALK lessons. This creates a learning environment that, most likely, is more beneficial for student learning. However, how FALK influences the learning environment and learning is a question for future research.

The qualitative results also reveal challenges (barriers) mostly linked to the teachers’ work situation, working time and practical challenges in combining PA and teaching. Time constraints and competing demands to meet the curriculum are highlighted in previous research [17, 31, 32]. In our study, competing demands are not so prominent, but a few teachers perceive FALK as ‘controlling’ because pulse-raising PA needs to be combined with theoretical teaching. This kind of challenge could possibly be solved with the help of other teachers finding suitable FALK lessons for the subject concerned. However, the teachers’ work situation needs to be considered when deciding to implement FALK in schools. As with any organisational change, it is important to have a dialogue and involve those affected by the change, which in the long run paves the way for successful implementation [33].

The qualitative results also point towards possible improvements in FALK, with the teachers emphasising the need for even more consensus on the method and better coordination of work materials. Improvements involve better communication in the teaching team and organising work materials more clearly by subject and grade. We believe these improvements are ‘low-hanging fruits’ and are relatively easy to improve. Furthermore, the need for improvements will most likely emerge when FALK is tested on a larger scale in more schools with varying preconditions.

Finally, we want to discuss FALK in relation to the school leader role and sustainability. Leadership, organisational support, and resources are factors commonly reported on in implementation research [34], as well as research on implementation of school-based health interventions [35, 36] and successful implementation of lesson-integrated PA [17]. Even though the school leader’s role was not evident in our results, it was fundamental for setting up our researcher-teacher collaboration and for providing resources and thereby creating good conditions for staff to put FALK into practice. As indicated in previous research, attitudes among school leaders are crucial for implementing health-promoting initiatives in schools in general [37], as well as for providing resources and creating structures and processes for lesson-integrated PA to be sustainable over time [14, 15]. Sustainability is an urgent research task in school-based health interventions, and this also applies to FALK to become part of organisational routines in schools and result in long-lasting effects on the children’s total PA levels [35, 38].

### Strengths and limitations

We consider the mixed methods approach a strength of the study as the explanatory sequential design contributes to a more in-depth understanding of the effectiveness and implementation of the FALK intervention. Below, we discuss the strengths and limitations of the included quantitative and qualitative approaches.

The study was conducted on a limited number of students and with only two primary schools involved. The relatively small size of the study and the fact that schools in Sweden have varying preconditions means that the generalisability of the study is somewhat limited. Another limitation of the study could be the quasi-experimental design, i.e. individual students were not randomised to the intervention group or the control group. For obvious reasons, it is difficult to randomise students when the groups are located in different schools. The fact that the intervention and control groups were in different schools can also be considered a strength of the study because the groups did not affect each other (i.e. there was no ‘spillover effect’).

Different methods can be used to objectively measure PA. Pedometers measure the number of steps while accelerometers measure changes in the speed of movement. The advantages of pedometers used in the present study are that participants can monitor their own activity progress, and pedometers are suitable for use in interventions. On the other hand, a disadvantage of the ‘number of steps’ measure is that it does not tell us anything about intensity, but pedometers can still be used to measure an individual’s total PA over time. An advantage of the accelerometer is that in addition to total PA, it also shows intensity, duration, and frequency. A disadvantage of the accelerometer is its price. Both pedometers and accelerometers are insensitive to activities such as swimming, cycling and arm movements. Nevertheless, both devices can provide a good picture of total PA [39, 40].

It is common practice to report the results of intervention studies, such as the evaluation of a new drug or a manual-based programme, with a detailed description of the methods so that other researchers can repeat (replicate) the study. The FALK method has to be adapted to the different conditions in different schools and is therefore difficult to describe in detail, using a step-by-step approach. The core of FALK is to integrate PA into theoretical, compulsory lessons. In addition to the influence of the level of knowledge and maturity of the students, the implementation of FALK is also influenced by the individual school’s staffing resources, the availability of outdoor activities, the composition of the student group, the group dynamics, the preferences of students and teachers, and the current weather conditions.

The FALK lessons in the present study are designed for students in grades 1–3, i.e. children aged 7–9 years (see Additional file 1). For other age groups, the content of the lessons needs to be adapted. The proportion of students with insufficient PA increases in higher grades [3], which motivates the development of school-based methods that increase PA also in older students.

A strength of the interview study is that it explores enablers and barriers of lesson-integrated PA from three perspectives. The interviews with students, parents and teachers contribute to a more nuanced and credible picture of the implementation process [23], and it is a strength that the voices of students are heard because they are the ones participating in the FALK lessons.

A limitation is that the study only reflects experiences from one medium-sized municipal primary school in Sweden. At the same time, the study’s results can be transferable to similar methods (interventions) within primary schools given that contextual conditions are considered [18, 23]. Finally, a limitation is the short and non-recorded interviews. A consequence of this approach is that it provides a more limited interview material compared to audio-recorded interviews, which can affect the depth of the qualitative analysis. Another risk is bias, with notetaking being influenced by the researcher’s preunderstanding and interpretations. We have handled this risk by having interviews and research questions covering both enablers and barriers with FALK, as well as being several authors involved in both taking notes and the analysis. However, the difficulty of capturing all the details when taking notes should be taken into account. Considering that the interview questions were straightforward (what was good, less good and suggestions for improvement) and did not touch on sensitive issues, we still deemed it sufficient and pragmatic to document the interviews through notes. A recent review shows that rapid (interview) methods, despite their limitations, can be an alternative to traditional qualitative methods [41].

### Future research and practical implications

We have four suggestions for future research in addition to investigating the sustainability of FALK. First, we suggest investigating FALK effectiveness in older age groups and under varying circumstances, meaning schools located in both high and low socioeconomic areas. This is important as we know from previous research that a student’s PA follows a social gradient, with students from high-income areas having higher levels of PA [3, 10]. Second, to explore the effectiveness of FALK for students with special needs, and to study the implementation of FALK in special needs education and what kind of adaptations are needed. Third, although FALK is not a manual-based method, the balance between fidelity and adaptation in varying school contexts needs further study [42]. Fourth, to explore how FALK influences the learning environment in the schools and how FALK influences the student’s learning.

Under the right circumstances, the practical implication of FALK is that it is a method worth trying. FALK does not require extensive financial investment, extra facilities, extra school staff or lessons outside the regular schedule. However, what is needed is the courage to think ‘outside the box’ in teaching– implying that teaching can take place in other ways than sitting indoors in a classroom. Also needed are acceptance among teaching staff, and support from the school leader.

## Conclusions

We conclude that FALK is a useful and feasible method for integrating PA into theoretical teaching. FALK effectively increases the average number of SPD and reduces the number of students not reaching the recommended level of PA. Moreover, FALK is experienced as a positive, clear, and flexible method encouraging PA and concurrent learning. FALK also contributes to professional development, collegial learning, and collaboration among teachers. Challenges experienced concern the teachers’ work situation, time, finding suitable learning activities, outdoor school environment changes, and extreme weather conditions. Suggested improvements in FALK include consensus on the way of working among teachers, and better organisation of work materials. The results taken together, we conclude that FALK is worth testing at more schools, given that implementation and sustainment of FALK considers both general enablers and barriers, as well as context-specific factors at the individual school.

### Electronic supplementary material

Below is the link to the electronic supplementary material.

Supplementary Material 1

## Data Availability

The datasets used and analysed during the current study are available from the corresponding author on reasonable request.

## Declarations

### Authors’ information

The first author (RL) is a senior lecturer in public health sciences with experience in health promotion, implementation, evaluation, and qualitative research. The second (EL) and third (SJ) authors are teachers working in a primary school in Borlänge municipality, Sweden. EL is a registered teacher with over 38 years of experience in teaching physical education and health. SJ is also a registered teacher with 16 years of teaching experience and with an interest in PA. The fourth author (TL) is a licensed physician by profession and an associate professor and senior lecturer in public health sciences with extensive experience in teaching and research on lifestyle factors, especially PA, and health.

## Abbreviations

FALK: Swedish abbreviation for ‘Physical Activity and Lesson in Combination’
MI: Movement integration
PA: Physical activity
SPD: Steps per day
