# Supplementary material for: ‘We get to learn as we move’: effects and feasibility of lesson-integrated physical activity in a Swedish primary school
Source: BMC Public Health. 2024 Apr 19;24:1087. doi: 10.1186/s12889-024-18509-7 (PMC11027527; doi:10.1186/s12889-024-18509-7)
Supplement: Supplementary file 1 — Supplementary Material 1 [file 12889_2024_18509_MOESM1_ESM.docx]

**Descriptions of FALK lessons**

**Background information**

There is no detailed manual for FALK lessons, and how to conduct lessons can vary depending on school conditions. The most important thing, and the core of FALK, is to conduct lessons with PA in combination with a theoretical subject. However, ‘in combination with’ should not be over-interpreted, and the students do not have to exercise PA and the theoretical subject at the exact same time. Still, PA should be integrated into the lesson, resulting in active lessons.

The five FALK lessons we outline below come from an extensive compilation of ideas continually expanded on by teachers at the intervention school. We like to emphasise that the lessons do not need to be complicated; both in the pilot study and in this study, we found that the students appreciated the set-up and simply found the lessons fun!

**Reading comprehension**

Preparation

The teacher writes sentences that fit the level of knowledge in the class and that describe an object (e.g., ‘A red, green, yellow and blue bird with a curved beak’) on a piece of paper. The teacher also creates a picture that corresponds to the respective sentence.

Performance

Each student is given a sentence, reads it, and then runs about 20 metres away to the place where the pictures are laid out. The student gets the picture that fits the sentence, runs back to the starting point with it and shows it to the teacher (see below picture). After that, the student runs and puts the picture back, runs back to the starting point again and is given a new description. The exercise can be conducted in pairs if one in the pair is very weak in reading.


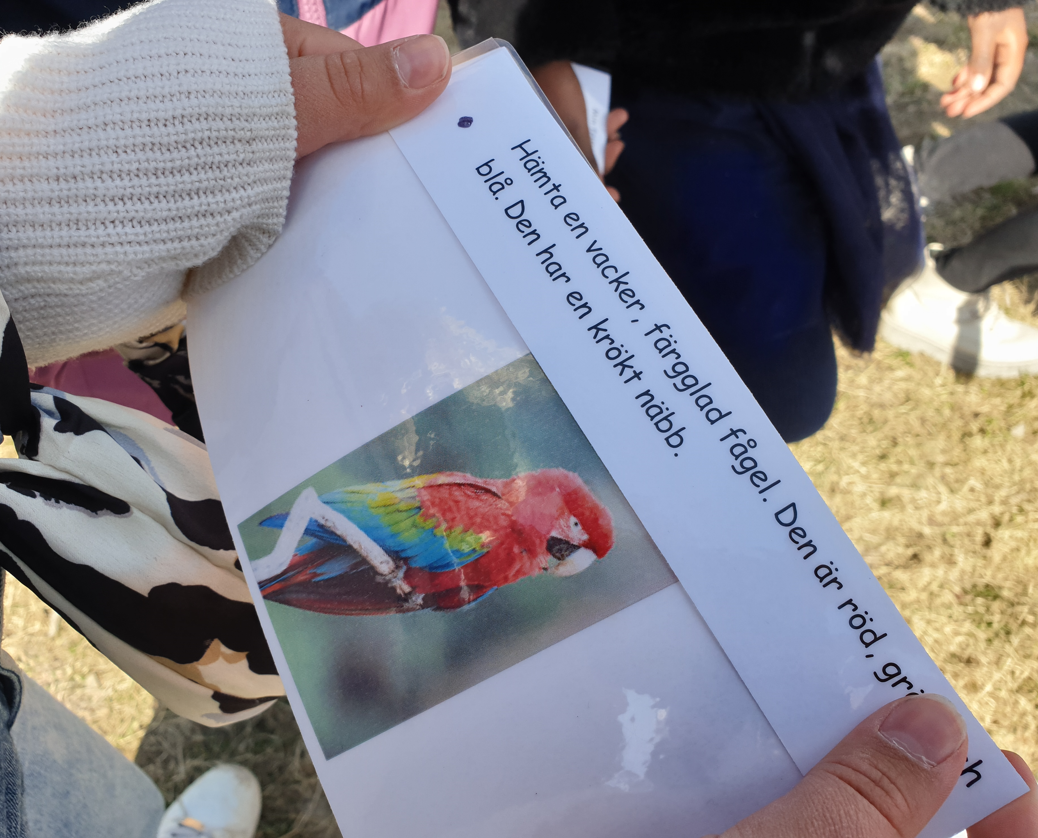


**‘Spider exercises’**

Preparation

Write down questions that are suitable for your class, and in subjects, you have studied recently in school. These can be mixed knowledge questions in different subjects, and can also be questions with or without response options. Place the question in a small plastic pocket and tie a string to the plastic pocket so that it can be hung up. Place the questions in your immediate environment 20 - 50 metres from the starting point.

Performance

A teacher or teaching assistant (hereafter teacher) stands at the chosen starting point, for example in the middle of the football field or in a glade in the forest. Instruct the students that they will run and answer as many questions as possible, one question at a time. The questions can be done in any order. The students run out and choose a question, read the question and think about the answer on the way back to the teacher at the starting point. They tell the answer to the teacher and if it is the right answer, the student runs to the next question. Students can also work in pairs. They should always return to the starting point before taking the next question.

**Yes and no questions**

Preparation

Write questions that are about something that you are reading right now in class (e.g., outer space, farm animals, Vikings). The questions should be formulated so that it is only possible to answer, ‘Yes’ or ‘No’. If you want to reuse the questions, it is a good idea to either laminate them or put them in a plastic pocket.

Performance

A teacher asks a question, for example, ‘Is a young pig called a lamb?’. The students respond by making one movement for ‘Yes’ and another movement for ‘No’. An alternative is to use a coloured cone that symbolises ‘Yes’ and a cone in another colour that symbolises ‘No’. Determine a starting point where all the students will gather before the question is asked. The teacher calls out a question and the students respond by running to the appropriate cone. It is extra fun if the students move in different ways; backwards, on tiptoe, on their heels, etc.

**Spelling walks**

Preparation

Words that are difficult to spell, and that fit the level of knowledge in the class, are written down on a piece of paper. If you want to reuse the words, it is a good idea to either laminate them or put them in a plastic pocket.

Performance

The teacher goes first, and the students walk two by two after each other. Each pair of students has a note with the words to be practised and take turns questioning each other while moving forward. The students spell out the word for their mate. When the word is spelt correctly, the students take turns with reading and spelling.

**Maths lesson with skipping rope**

Preparation

Prepare mathematical problem-solving tasks adapted to the students’ age, then laminate them or put them in a plastic pocket. Divide the class into groups with 3-4 students in each. Two skipping ropes per group are needed.

Performance

Each group should solve the mathematical problems while keeping the skipping ropes moving. The group members are allowed to exchange tasks with each other, but everyone should both solve math tasks and jump.
